# Disparate Recruitment and Retention of Plasmacytoid Dendritic Cells to The Small Intestinal Mucosa between Young and Aged Mice

**DOI:** 10.14336/AD.2021.0119

**Published:** 2021-08-01

**Authors:** Rosemary A Hoffman, Sulan Huang, Geetha Chalasani, Abbe N Vallejo

**Affiliations:** ^1^Thomas E. Starzl Transplantation Institute,; ^2^Department of Surgery,; ^3^Department of Health Promotion and Development,; ^4^Division of Renal-Electrolyte, Department of Medicine,; ^5^Division of Pediatric Rheumatology, Department of Pediatrics, University of Pittsburgh,; ^6^Division of Rheumatology, UPMC Children’s Hospital of Pittsburgh, Pittsburgh, PA 15224, USA.

**Keywords:** adoptive transfer, chemokine, endotoxemia, inflammation, intestinal intraepithelial cell, plasmacytoid dendritic cell

## Abstract

Plasmacytoid dendritic cells (pDC), a highly specialized class of innate immune cells that serve as rapid sensors of danger signals in circulation or in lymphoid tissue are well studied. However, there remains knowledge gaps about age-dependent changes of pDC function in the intestinal mucosa. Here, we report that under homeostatic conditions, the proportion of pDC expressing C-C chemokine receptor 9 (CCR9) in the intestinal intraepithelial cell (iIEC) population is comparable between young (2-4 months) and aged (18-24 months) mice, but the absolute numbers of iIEC and pDC are significantly lower in aged mice. Employing the classic model of acute endotoxemia induced by lipopolysaccharide (LPS), we found a decrease in the proportion and absolute number of intraepithelial pDC in both young and aged mice despite the LPS-induced increased expression of the chemokine C-C ligand 25 (CCL25), the ligand of CCR9, in the intestinal mucosa of young mice. In adoptive transfer experiments, a significantly lower number of pDC was retained into the intestinal layer of aged host mice after LPS administration. This was associated with recoverable pDC numbers in the intestinal lumen. Furthermore, co-adoptive transfer of young and aged pDC into young hosts also showed significantly lower retention of aged pDC in the epithelial layer compared to the co-transferred young pDC. Collectively, these data show age-associated changes in mucosal CCL25 gene expression and in pDC number. These may underlie the reported inadequate responses to gastrointestinal pathogens during chronologic aging.

An effective intestinal mucosal immune system prevents pathogen invasion while simultaneously mediating tolerance to commensal organisms. During the aging process, the immune system is less efficient at controlling infections at mucosal surfaces [1]. Studies of the mucosal response to pathogen challenge in aging mice, including *Francisella tularensis* vaccine [2], *Mycobacterium tuberculosis* [3], influenza virus [4] and intestinal parasites [5] indicate altered migration of innate immune responder cells to the infection site, altered cytokine profiles and decreased production of high-affinity neutralizing antibodies. Clearly, multiple factors contribute to the reduced immune responsiveness of aged mice relative to that seen in young mice.

Since the immune response to infection relies on antigen presentation and effector functions of dendritic cells (DC), much of the efforts in aging research have focused on evaluating function of myeloid DC (mDC), the major class of DC. These cells, however, are ontogenetically and transcriptionally regulated differently from plasmacytoid DC (pDC). Indeed, pDC comprise of highly specialized DC of non-myeloid origin that serve as more rapid sensors of infection and other danger signals [6-8]. Both pDC and mDC undergo changes with advancing age. In humans, discordant observations have been published regarding absolute numbers of peripheral blood pDC and classical monocyte-derived mDC [9-11]. Other studies demonstrated overall decreased DC function with aging, including reduced production of interferon (IFN)-α and interleukin (IL)-12, impaired phagocytosis of antigen and migration in response to chemokines [12-14]. However, stimulation of aged blood-derived mDC with LPS can lead to higher production of tumor necrosis factor (TNF)-α and IL-6, and to enhanced response to endogenous antigens [15]. However, there is an overall defective signaling of toll-like receptors (TLR) in aged blood mDC that also correlates with a poor response to vaccination [16]. In mice, bone marrow-derived mDC from aged mice appear to have normal TLR-mediated immune responses [17]. Notably, aged mouse pDC have impaired TLR9 signaling due to defective activation of IFN-regulatory factor 7, a key regulator of the type I IFN pathway [18]; TLR9 being one of the distinguishing marker/receptor of pDC [6]. Other investigators have found altered splenic mDC function in aged mice, including decreased cytokine production, altered subset distribution and inability to upregulate costimulatory molecules upon TLR stimulation [19]. All of these studies reinforce the notion that the innate immune response during the aging process, including DC migration and cytokine production, contribute to less efficient immune protection in aged mice and humans.

Chemokines and their receptors play an important role in orchestrating the complex patterns of cell trafficking within the immune system during homeostasis as well as during the responses to antigenic stimulation. Inhibition of expression of chemokines results in decreased numbers of intraepithelial and lamina propria CD8^+^ T cells and also reduced numbers of intestinal eosinophils and macrophages [20]. Small intestinal epithelial cells constitutively produce chemokine C-C motif ligand (CCL)25 that attracts cells expressing chemokine C-C motif receptor (CCR)9, including B cells [21], T cells [22] and pDC [23]. In *CCL25^-/-^* and *CCR9^-/-^* mice there is a significant decrease in the ratio of T cells to epithelial cells and a decrease in the proportion of CD8^+^ T cells in the lamina propria [24]. There is also impaired homing of adoptively transferred antigen specific CD8^+^ T cells to both the lamina propria and epithelial layer of *CCL25^-/-^* mice [24]. These data indicate that CCR9-CCL25 interaction plays an important role in maintaining various cell populations in the intestinal mucosa.

In the present work, we examined whether expression of mucosal CCL25 correlates with the homing of pDC to the intestinal mucosa of young and aged mice. Under homeostatic conditions, we examined the numbers of intestinal intraepithelial cells (iIEC) and pDC in young and aged mice. Under inflammatory conditions with challenge to LPS, a proven model of acute endotoxemia in a variety of experimental settings including during aging [25-28], we examined the patterns of CCL25 expression and the ability of adoptively transferred pDC to migrate to the intestinal epithelium and subsequently into the lumen. Considering the central role of pDC in both innate and adaptive immunity, we reasoned that access of pDC to both the epithelial and luminal compartments ultimately influences mucosal immunity, if not tolerance, to the microflora therein.

## MATERIALS AND METHODS

### Animals

Research involving animals were performed according to protocols approved by Institutional Animal Care and Use Committees of the University of Pittsburgh. Young (6-8 weeks of age) and aged (18-24 months) C57BL/6 (B6) mice were obtained from the National Institutes of Aging and Charles Rivers Laboratories. Some of the mice used in this study were aged at the University of Pittsburgh under specific pathogen free (SPF) conditions, but not *Helicobacter*-free conditions. Confirmed *Helicobacter*-free aged mice purchased from Charles River were housed in *Helicobacter*-free SPF conditions along with the young mice used in the same experiments. B6.129P(Cg)-*Ptprc^a^ Cx3cr1^tm1Litt^*/LittJ were obtained from Jackson Laboratories, housed in SPF conditions and used to detect expression of C-X3-C motif chemokine receptor 1 (CX3CR1) on pDC in the intestine.

### Flow Cytometry

iIEC and intraluminal cells (iLC) were identified by immunostaining with following fluorochrome-conjugated antibodies: CD3e (145-2C11), B220 (RA36B2), class II (M5/114.15.2), CD11b (M1/70), CD11c (N418), PDCA-1 (eBio927), CD45.1 (A20) and CD45.2 (104) from eBiosciences; Ly6C (AL-21) and CD103 (M290) from BD Biosciences; CCR9 (242503) from R&D Systems. Unfixed cells were analyzed using an LSR II (BD Biosciences) to collect raw cytometric data. Off-line analyses of cell populations were performed using either FACS Diva (BD Biosciences) or FlowJo software (Tree Star). Instrument calibration, construction of off-line compensation matrices to discriminate between background and true fluorescence signals, and electronic gating of cell populations of interest followed previously established procedures [29, 30].

### LPS-induced intestinal inflammation.

Young and aged mice were injected intraperitoneally (IP) with 2.0 mg LPS (Sigma) per kg body weight 18-20 hours before harvesting tissues for analysis. This low dose of LPS was empirically established a higher dose is acutely lethal to aged mice, corroborating work by other investigators [31, 32]. As a second inflammatory system, mice were also orally gavaged with 10 µg of cholera toxin (Sigma) in 0.3 ml carbonic buffer (0.1M NaHCO_3_) 2-3 hours before harvesting tissues for analysis [23, 33].

### Isolation of iLC and iIEC

The intestines were removed from euthanized mice and placed in medium on ice. For iLC harvest, the intestine was flushed with cold RPMI medium containing 5% fetal calf serum (FCS) to remove intestinal contents and then pipetted vigorously before filtering through nylon mesh. The filtrate was washed by slow speed centrifugation, resuspended in 5 ml of the FCS-RPMI medium, and subjected to isopycnic centrifugation over lympholyte-M density gradient to recover cells from the interface.

Standard techniques for obtaining iIEC were employed [34]. Briefly, the intestine was flushed to remove intestinal contents, the Peyer’s Patches extracted, and the intestinal tube opened and cut into 10-20 mm pieces. The tissue was suspended in PBS containing 10% FCS, 1 mM ethylenediaminetetraacetic acid (EDTA), and 1mM diothiothreitol (DTE) and stirred at 37°C for 45-60 minutes. The supernatant was filtered through nylon mesh, the tissue pieces returned to phosphate buffered saline (PBS) containing FCS-EDTA-DTE, vigorously vortexed to remove the epithelial layer and filtered through nylon mesh to remove tissue. The cells were centrifuged, resuspended in cold RPMI and run through a loosely packed nylon wool column. The eluate was centrifuged, the cells subjected to a 44% - 67% Percoll gradient centrifugation and cells recovered from the interface were counted and phenotyped by flow cytometry.

### Adoptive transfer

Young CD45.1 congenic mice or aged B6 CD45.2 mice were injected IP with a daily dose of 10 μg Fms-like tyrosine kinase 3 ligand (Flt3L) for 10 days. Splenocytes were phenotyped cytometrically and 3 to 5 x 10^7^ splenocytes (containing 0.8 to 1.5 x 10^6^ Ly6C^+^, B220^+^ pDC) were injected into the tail vein of recipient syngeneic mice. After 18-20 hours, iIEC were harvested and the proportion and absolute numbers of CD45.1^+^ cells were determined by cytometry. In the co-transfer of young and aged pDC experiments, the aged mouse splenocytes were phenotyped and stained with the vital dye carboxyfluorescein succinimidyl ester (CFSE; Invitrogen) before adoptive transfer so as to cytometrically visualize the transferred aged cells.

### Quantitative real-time polymerase chain reaction (RT PCR)

Ileal intestinal mucosal samples were harvested 18 hours after IP injection with LPS or PBS. RNA was isolated using TRIzol reagent (Invitrogen). Twenty ng total RNA of each sample was reverse transcribed into cDNA using High Capacity cDNA Reverse Transcription kit (Applied Biosystems). Reverse transcription was performed 10 min at 25°C, 120 min at 37°C, 5 sec at 85°C in a thermal cycler according to manufacturer specifications.

All primers and probes (Assays on Demand) were purchased from Applied Biosystem. We quantified transcripts of 18S as the housekeeping gene. A reference RNA sample prepared from control mouse intestinal mucosa was included in each assay as a calibrator. PCR was performed for 40 cycles of denaturation for 15s at 95°C and annealing and extension for 1 min at 60°C, using an ABI Prism 7900 sequence detection system (Applied Biosystems). Relative expression of the RT-PCR products was determined using the standard ∆∆CT method.

### Western blot

Ileal mucosal scrapings were lysed in ice-cold RIPA buffer supplemented with protease inhibitor cocktail (Sigma), protein concentrations determined using Bradford protein assay (BioRad) and 30 μg of protein was added per lane to an SDS-polyacrylamide gel. Binding of CCL25 antibody (Santa Cruz Biotechnology) followed by secondary anti-rabbit horseradish peroxidase antibody (Santa Cruz Biotechnology) was detected using SuperSignal West Pico Chemiluminescent substrate (Pierce Biotechnology).

### Enzyme-linked immunosorbent assays (ELISA)

Ileal mucosal scrapings were homogenized in a protease inhibitor mixture (Calbiochem), centrifuged at 10,000 rpm for 10 min. and supernatants serially diluted for analysis using CCL25 ELISA kit (R&D Systems). Protein concentration was determined with a BCA protein assay kit (Pierce) and the amount of CCL25 was expressed as ng CCL25/mg of protein.

### Statistical analysis

Data were analyzed by Kruskal-Wallis analysis of variance (ANOVA). Between group comparisons used *post hoc* Dunn’s statistic from ANOVA. Unpaired Student’s t test was used when 2 groups were compared. A P-value < 0.05 was considered statistically significantly different.

**Figure 1.**
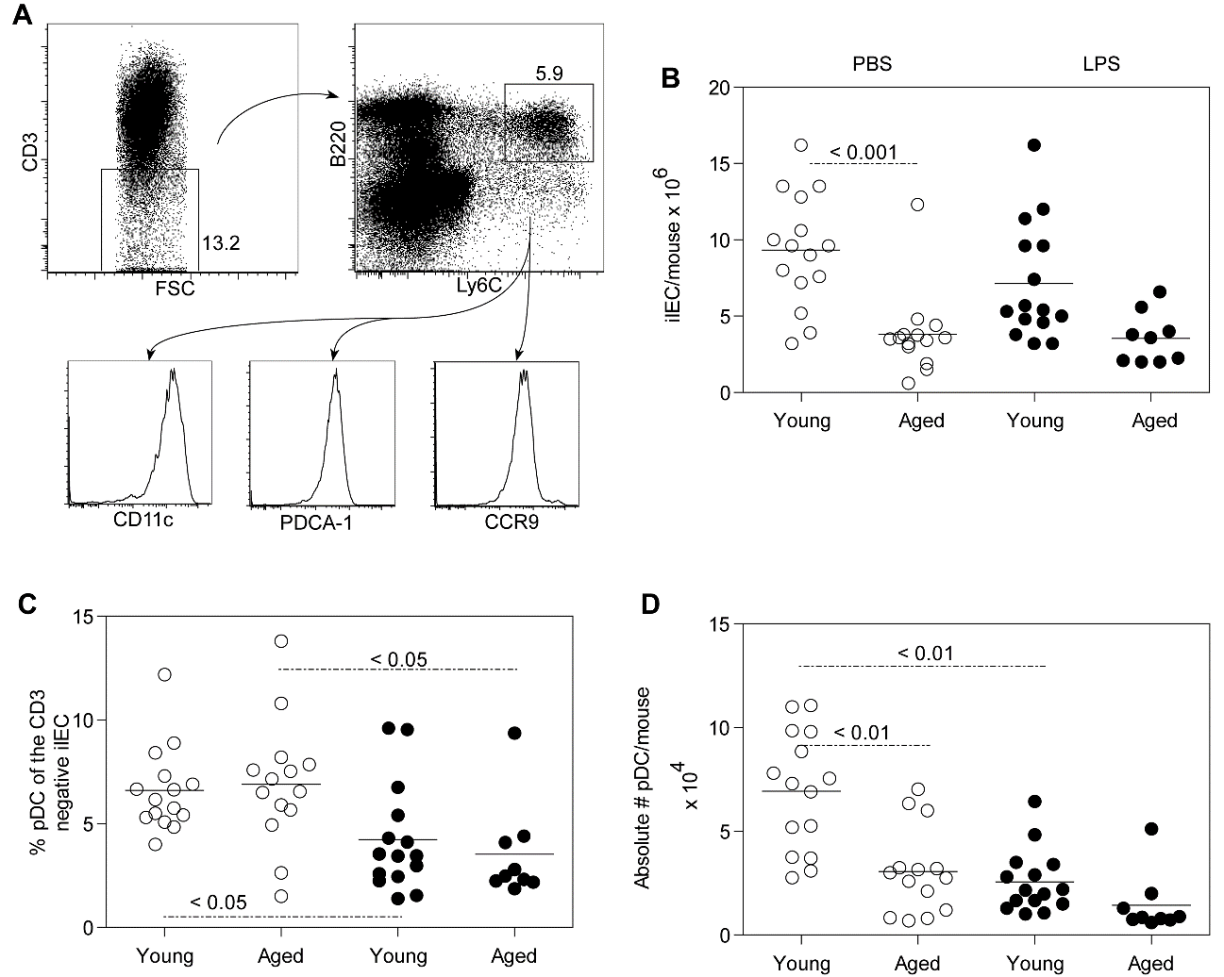
Characterization of pDC in the intestinal intraepithelial cell (iIEC) population. The CD3^-^ iIEC population was gated and pDC were characterized as Ly6C, B220, CD11c, CCR9 and PDCA-1 positive (A). The absolute number of iIEC (B) proportion (C) and absolute number (D) of pDC per intestine of young and aged PBS and LPS treated mice ae depicted (9-15 mice per group). Each data point represents results from one mouse, the open circles are PBS treated and the closed circles are LPS treated mice. The horizontal bars in the graph indicate mean values. The P value indicated in the figure was calculated by ANOVA.

## RESULTS

### Small intestinal intraepithelial pDC from young and aged mice

The absolute number of iIEC, and the proportion and absolute number of pDC recovered from the epithelial layer of young and aged mice 18 hours after administration of PBS or LPS was determined. The gating strategy for small intestinal intraepithelial pDC, depicted in Figure 1A, was to gate on the CD3^-^ iIEC population, followed by gating on Ly6C^+^, B220^+^ cells and confirmed by expression of CD11c, PDCA-1 and CCR9 following a previously described procedure [23]. Additional phenotype data (Supplemental Fig. 1) indicated that the Ly6C^+^, B220^+^ population was class II^+^, CD11b^-^, CX_3_CR1^int^ and CD103^lo/-^.

**Figure 2.**
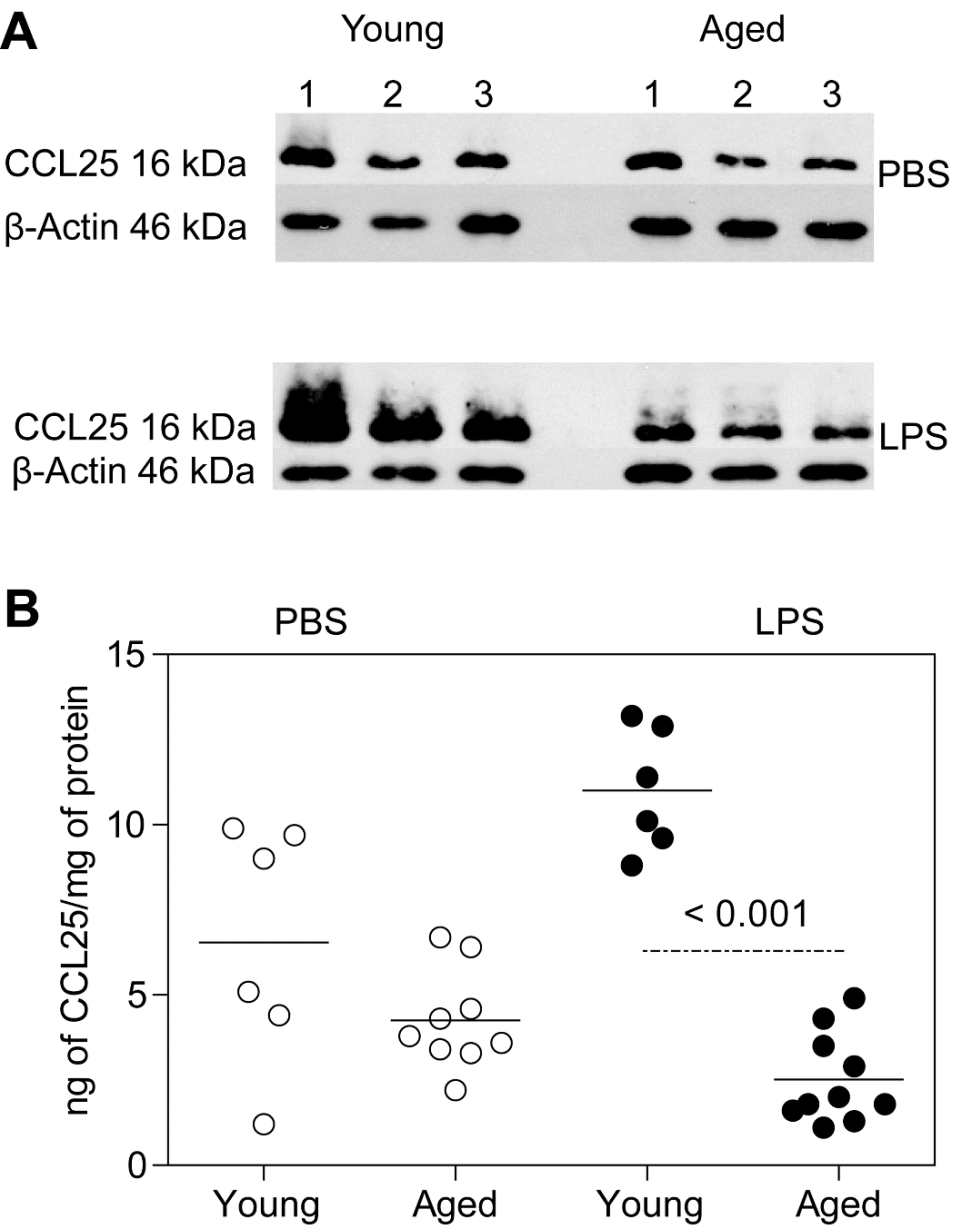
Expression of CCL25 protein in small intestinal mucosal lysates. Young and aged B6 mice were injected IP with LPS (2.0 mg/kg) or PBS, ileal mucosal samples were harvested 18 hours later and processed for quantitation of CCL25 protein levels by Western (n = 3/group) (A) and ELISA (n = 6-10/group) (B). Three individual young and aged PBS- and LPS-treated B6 mice are depicted in the Western blot and 6-10 mice per group were analyzed by ELISA. Scatter plots and P values in b were determined as in Figure 1.

The absolute number of iIEC recovered from aged mice was significantly less than that recovered from young mice injected with PBS (P < 0.001, Fig. 1B). Following LPS administration, lower numbers of iIEC were also observed in aged mice compared to young mice, although the difference was not statistically significant. Additional data on the proportion and absolute number of CD3^-^ iIEC in young and aged mice are shown in Supplemental Figure 2. However, the proportion of pDC in the CD3^-^ iIEC population was significantly decreased (P < 0.05) after LPS administration in both young and aged mice (Fig. 1C). The number of pDC recovered from LPS-treated young mice was also significantly decreased (P < 0.01) compared to PBS-treated young mice (Fig. 1D). All these data demonstrate that aged mice have significantly less iIEC and pDC than young mice with a significant fluctuation in proportion and absolute number of pDC in the intestinal mucosa in response to a systemic inflammatory stimulus.

**Figure 3.**
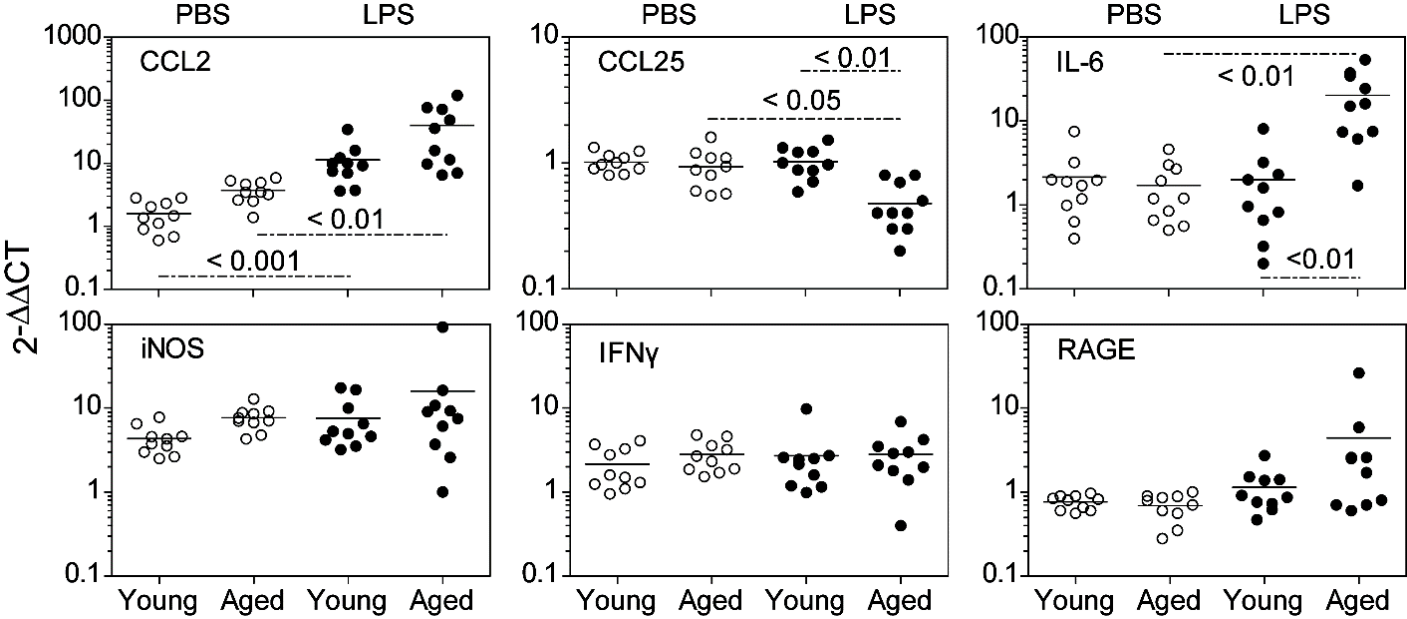
Expression of mRNA for chemokines and inflammatory mediators in the intestinal mucosa. mRNA levels of CCL*2*, *CCL25*, *IL-6*, *iNOS*, *IFN*γ and *RAGE* were assayed by real-time RT-PCR on mucosal lysates 18 hours after IP injection of PBS or LPS in young and aged B6 mice (n = 10/group). Results are expressed relative to a reference mRNA mucosal sample run with each assay. Scatter plots and P values were determined as in Figure 1.

### CCL25 expression in the intestinal mucosa

Expression of CCR9 on pDC is required for their migration to the small intestinal mucosa in response to its chemokine ligand, CCL25 and the epithelial layer of *CCR9^-/-^* mice has been found to contain few pDC [23]. Therefore, we determined the amount of CCL25 protein in intestinal ileal mucosal scrapings of young and aged mice. Western blot data in Figure 2A from three individual young and aged mice show that CCL25 levels in PBS-treated young and aged mice were equivalent. After LPS administration however, young mice expressed more CCL25 protein than aged mice. Quantitative ELISA data in Figure 2B also show no significant differences in CCL25 levels between young and aged PBS-treated mice, although young mice had variable amounts of CCL25. In contrast, LPS administration resulted in significantly increased CCL25 levels in young mice compared to aged mice (P < 0.001). In fact, aged mice had even lower mean levels of CCL25 following LPS administration than those treated with PBS, indicating a failure to upregulate CCL25 after LPS administration. However, even in young mice, neither the proportion nor absolute number of pDC correlated with the increased levels of CCL25 seen after LPS administration. Similarly, in the aged mice, decreased expression of CCL25 did not result in significant decreases in absolute pDC number compared to young mice.

We also examined whether iIEC numbers and CCL25 levels were similarly affected in another model utilizing cholera toxin, which is considered a rapid inducer of intestinal inflammation [23, 33]. In this case, the toxin was administered by oral gavage and the intestines were analyzed after 2-3 hours. Supplemental Figures 3A and B show that while the absolute number of iIEC per mouse significantly decreased in aged mice compared to young mice, the absolute numbers of pDC were not different between the two age-groups. Further, the levels of CCL25 were also not significantly affected by cholera toxin treatment (Supplemental Fig. 3C). We have observed optimal upregulation of CCL25 within 12-24 hours after LPS administration (unpublished empirical data). Thus, the 2-3 hours’ time frame for the cholera toxin model, wherein mDC are known to migrate from the lamina propria to the mesenteric lymph node [33], may not be optimal for studying pDC migration and the corresponding requisite CCL25 upregulation.

### Inflammatory gene expression profile of intestinal mucosa

The diminution of CCL25 protein expression in aged mice compared to young mice following LPS administration, prompted an examination of the expression of other mediators. RT-PCR for specific transcripts of *CCL2*, *CCL25*, *IL-6*, inducible nitric oxide synthase (*iNOS*), *IFNγ* and receptor for advanced glycosylation end product (*RAGE*) were performed. Data in Figure 3 show that *CCL25* mRNA levels in LPS-treated aged mice were significantly decreased compared to either young LPS-treated mice or aged PBS-treated mice. *CCL25* mRNA levels in young LPS-treated mice did not parallel the increase in CCL25 protein levels (Fig. 2). The basis for this observation is unknown, albeit another study has shown that LPS does not increase *CCL25* gene transcription [35].

In contrast to *CCL25* mRNA, Figure 3 also shows increased expression of transcripts for *CCL2*, *IL-6*, *iNOS* and *RAGE* in LPS-treated aged mice, although only the increases in *CCL2* and *IL-6* mRNA were significant (P < 0.01). Young mice showed a significantly increased level of *CCL2* mRNA (P < 0.001), but the levels of the other gene transcripts remained unchanged after LPS treatment. Interestingly, mRNA levels of *CCL2* and *iNOS* in the PBS-treated control were higher in aged mice than younger mice, although the changes were not significant. These data show that while mediators that promote inflammation, such as CCL2 and IL-6, were increased in the aged intestine, both mRNA and protein levels of the chemokine CCL25 were uniquely and significantly decreased. These were commensurate with a corresponding decrease in the number of pDC (and other iIEC) recovered from the aged intestinal mucosa.

**Figure 4.**
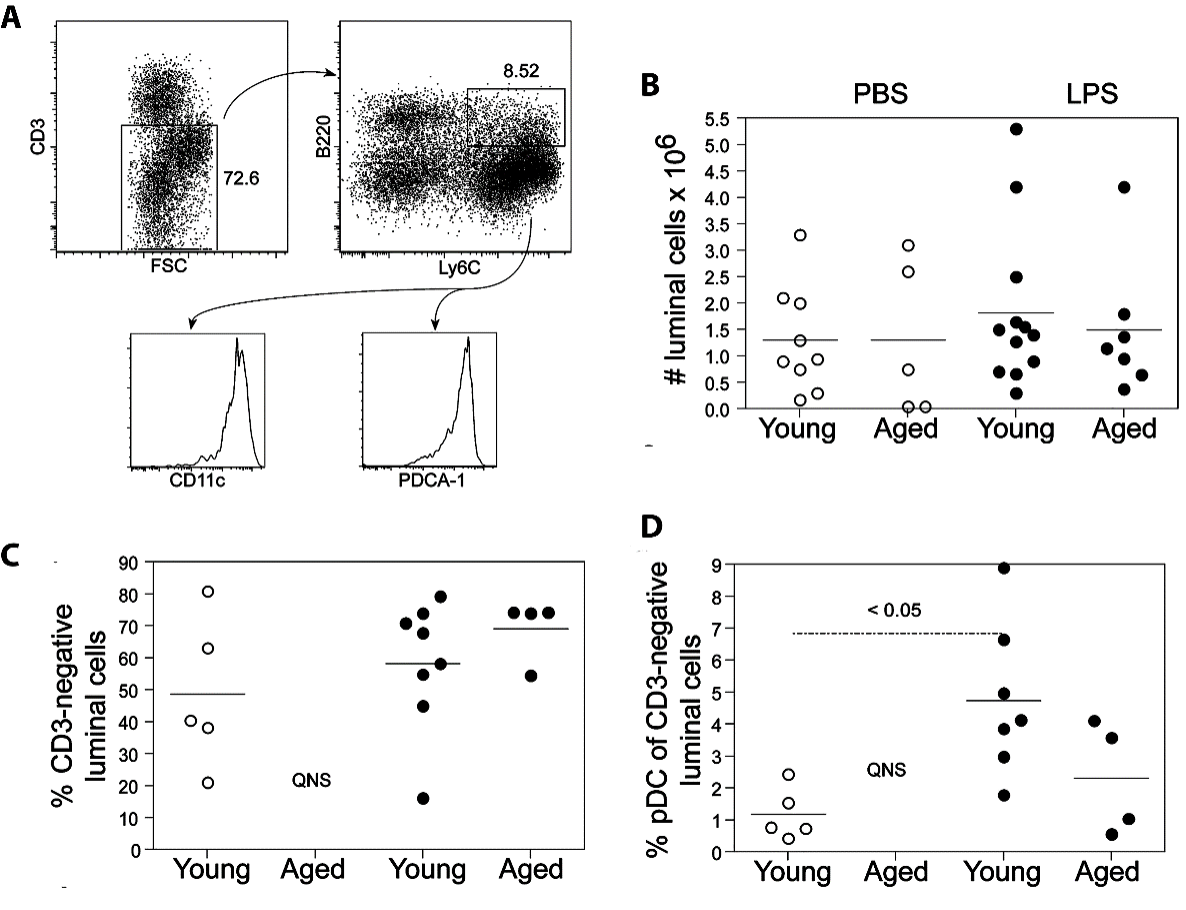
Characterization of cells recovered from the intestinal lumen. Small intestines were flushed with medium and cells recovered after a gradient centrifugation were counted. The CD3^-^ iIEC population was gated and pDC were characterized as Ly6C, B220, CD11c and PDCA-1 positive (A). The absolute number of intestinal luminal cells (iLC) recovered (B) (n = 5-11/group), the % of CD3 negative cells (C) and the % Ly6C^+^, B220^+^ pDC in young and aged mice (D) (n = 4-8/group) after administration of PBS and LPS were determined. There was an extremely low number of iLC from aged PBS treated mice. Scatter plots and P values were determined as in Figure 1. In (C) and (D), the number of cells recovered from the PBS treated aged mouse was insufficient for accurate analysis, is indicated by QNS and was excluded from statistical analyses.

### iLC of young and aged mice

One possible explanation for the decreased proportions of pDC in the epithelial layer of LPS-treated mice was pDC (and other iIEC populations) could extravasate into the gut lumen. The phenotyping strategy of iLEC populations is shown in Figure 4A. There were overall low numbers of total iLC recovered particularly in aged mice, but there was no statistical difference between the two groups regardless of LPS-treatment as shown in Figure 4B. When the iLC population were gated for CD3^-^ cells (Fig. 4C) and the corresponding CD3^-^ pDC subset (Fig. 4D), such cells were negligibly recovered from PBS-treated aged mice. There was however an LPS-induced increased number of recoverable CD3^-^ iLC in LPS-treated old mice (Fig. 4C). This was in marked contrast to young mice in which there were equivalent, non-statistically significant numbers of recoverable CD3^-^ iLC between PBS- and LPS-treated animals (Fig. 4C). With LPS, these iLC subsets were recovered from the aged mice, but were lower compared to young mice. While the mechanism of cell “movement” to the lumen is unknown, the number of iLC recovered could depend on how quickly they were eliminated from the lumen either by intestinal peristalsis or by bowel movement. Additionally, IP injection of LPS could be eliciting changes in intestinal physiology, in a manner analogous to that reported for gut microbiota-derived LPS [36, 37] thought to affect the number of iLC.

Phenotypically, iLC were all CD45^+^ indicating their hematopoietic origin. The iLC contained a higher proportion of CD3^-^ cells (Fig. 4C, 50-60%) than iIEC (Supplemental Fig. 2A, 11-14%). When gated on the CD3^-^ population, the Ly6C^+^, B220^+^ cells expressed high levels of CD11c and the pDC-specific antigenic identifier PDCA-1 (Fig. 4A), similar to those observed for pDCs within the IEC population (Fig.1A). In young mice, there was a significant increase in the proportion of pDC in the iLC obtained from LPS-treated compared to PBS-treated animals (Fig. 4D). Similar LPS-induced increase in recoverable pDC^+^ CD3^-^ iLC was observed in aged mice (Fig. 4D), similar the overall lower numbers of aged CD3^-^ iLC (Fig. 4C). These data indicate that during a systemic LPS-induced inflammatory response, aged pDC do not have detectable retention in the intestinal epithelial layer but are readily extravasated into the lumen. LPS-induced luminal extrusion of pDC may exacerbate the intrinsic loss of pDC during normal chronologic aging (as shown in Fig. 1D).

### Adoptive transfer of pDC from young mice into young and aged hosts

**Figure 5.**
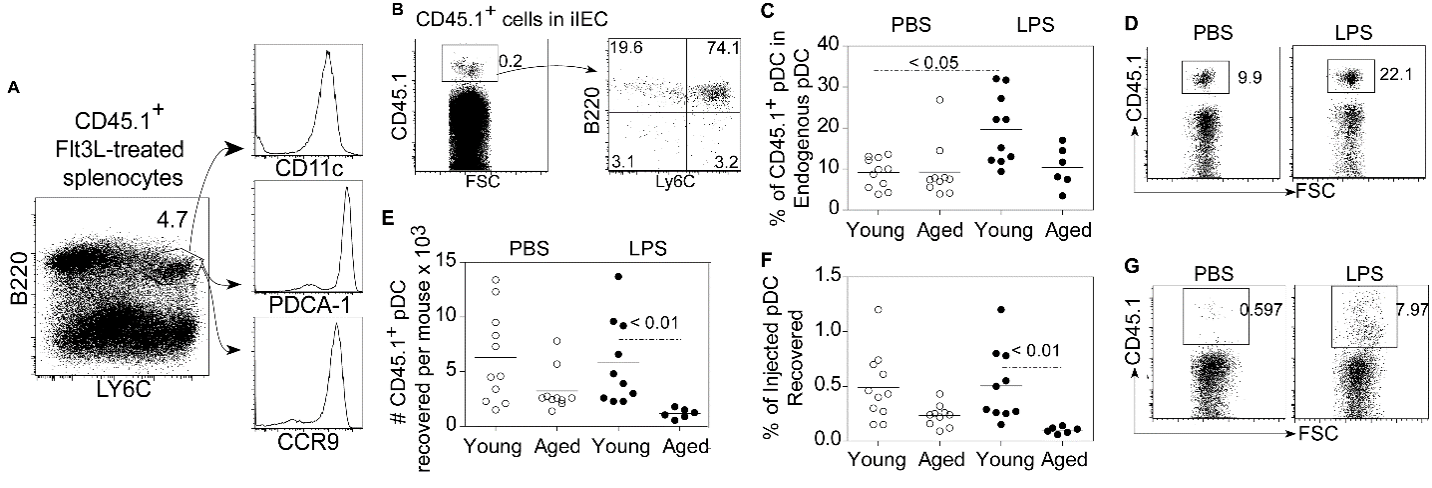
Adoptive transfer of congenic pDC. Splenocytes from young CD45.1^+^ Flt3L treated mice were phenotyped (A) and ~1 x 10^6^ pDC (Ly6C^+^, B220^+^) were adoptively transferred into young and aged PBS- and LPS-treated B6 mice. 74% of the CD45.1 cells recovered in the iIEC were LY6C^+^, B220^+^ (B). The proportion of CD45.1^+^ cells present in the pDC population within the iIEC of young and aged host injected with PBS or LPS was obtained by gating on pDC and determining the % of these cells that were CD45.1^+^ (C). A representative cytogram depicting the % CD45.1^+^ pDC in representative young PBS and LPS injected mouse pDC populations is depicted in D. The absolute number of CD45.1^+^ pDC recovered (E) and the % of injected pDC recovered (F) were determined. A cytogram representative of 3 separate experiments depicts the % CD45.1^+^ adoptively transferred cells present in the iLC of PBS and LPS injected young mice (G). Scatter plots and P values in C, E and F (n = 6-11/group) were determined as in Fig.1.

In order to determine if the CCL25 levels expressed in young and aged mouse intestine would influence the migration of adoptively transferred pDC, young CD45.1 mice were treated with recombinant Flt3L for 10 days to mobilize pDC from the bone marrow. Three-five x 10^7^ splenocytes (containing 2 to 5% Ly6C^+^, B220^+^, CD11c^+^, PDCA-1^+^, CCR9^+^ pDC) (Fig. 5A) were adoptively transferred by intravenous (IV) injection into CD45.2 hosts and the phenotype and number of donor-derived CD45.1^+^ cells recovered in the iIEC population was examined 16-18 hours later. The phenotype of the majority of CD45.1^+^ cells recovered from the epithelial layer was similar to the phenotype of the Ly6C^+^, B220^+^ cells in the Flt3L-treated spleen, indicating that the majority of the adoptively transferred splenocytes that reached the intestinal epithelial layer were the transferred pDC (Fig. 5B).

Expressing the adoptive transfer data as the proportion of adoptively transferred pDC present in the endogenous pDC population, Figure 5C shows a significantly increased proportion of adoptively transferred young pDC in young LPS-treated hosts compared to PBS-treated hosts. This increase in proportion of adoptively transferred pDC would be expected, since the endogenous pDC number was decreased in young mice after LPS administration (Fig. 1D). Representative dot plots of %CD45.1^+^ cells in the pDC population of young PBS- and LPS-treated mice are depicted in Figure 5D. In contrast, there was not an increase in the proportion of adoptively transferred young CD45.1^+^ pDC in aged hosts after LPS administration (Fig. 5C), even though there was a trend towards (but not statistically significant) decrease in the number of endogenous CD45.2^+^ pDC in aged mice after LPS administration (Fig.1D). The lack of a statistically significant decrease in the absolute number of endogenous CD45.2^+^ pDC in the aged LPS-treated mouse could be due to a failure of these cells to extravasate into the lumen, as such there may be limited space for occupancy and/or transit for transferred CD45.1^+^ cells.

Whether the data was expressed as absolute number of CD45.1^+^ pDC recovered or % of adoptively transferred CD45.1^+^ pDC recovered in the intestine, there was notable significantly fewer transferred cells that accumulated in the intestine of LPS-treated aged hosts than in the intestines of LPS-treated young hosts (P < 0.05; Fig. 5E and F). A similar absolute number/proportion of CD45.1^+^ pDC accumulated in young mouse intestinal epithelial layer whether or not the mice received LPS. All these data show decreased migration of transferred pDC to the intestine of aged hosts.

Figure 5G shows that a greater proportion of CD45.1^+^ cells were present in the iLC of LPS- versus PBS-injected mice (8% versus 0.6% CD45.1^+^ cells). These data reinforce the finding that under inflammatory conditions, pDC as well as other iIEC populations, extravasate into the lumen (Fig. 4). Significant numbers of adoptively transferred pDC were not present in either the spleen, intestinal lamina propria or the mesenteric lymph node of young or aged mice (data not shown).

Since Flt3L stimulates mobilization of pDC, we determined whether increased proportions of pDC entered the intestinal epithelial layer and lumen of FLt3L-treated mice (Supplemental Fig. 3A and B). The proportions of CD3^-^ cells in both iIEC (34.5%, Supplemental Fig. 3A) and iLC (87.6%, Supplemental Fig. 3B) populations were increased compared to iIEC (~11%, Supplemental Fig. 2A) and iLC (43%, Fig. 4C) of PBS-treated mice. Additionally, a greater proportion of Ly6C^+^, B220^+^ pDC (37.4%, Supplemental Figure S4a) was noted in the CD3^-^ iIEC population compared to the average of 6.5% seen in the iIEC of PBS-treated young mice (Fig.1C). The iLC population from the Flt3L-treated mouse also contained an increased proportion of pDC (8.5%, Supplemental Fig. 4B), compared to an average of 1% in young PBS treated mice (Fig. 4D). These data corroborate previous reports that pDC migrate from bone marrow to the intestinal epithelial layer [23], and our data indicate further movement of pDC from the epithelial layer into the lumen.

### Adoptive transfer of a mixture of young and aged pDC into young LPS-treated host

**Figure 6.**
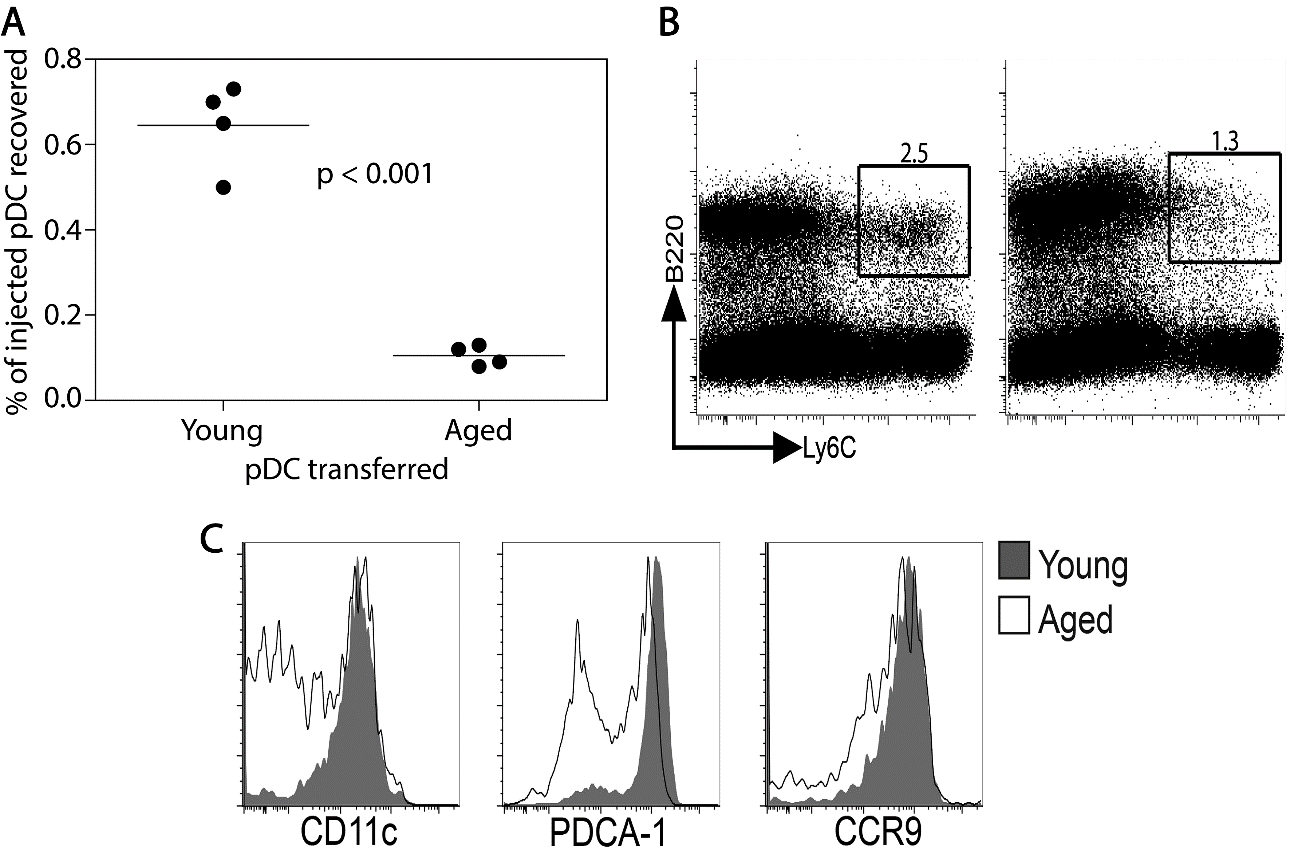
Comparison of migration of young and aged pDC to the epithelial layer of young mice injected with LPS. Splenocytes obtained from Flt3L-treated mice, containing an equal number of young (5.0 x 10^5^) and aged (5.0 x 10^5^) Ly6C^+^, B220^+^ cells were adoptively transferred into young LPS-treated mice. The number of young and aged pDC recovered from the epithelial layer was determined and the proportion of injected cells recovered was calculated (A). Control mice received 1 x 10^6^ young pDC or 1 x 10^6^ aged pDC. Recovery of young pDC was 0.74% and for the aged pDC were 0.18% of injected cells in these control mice. P value was calculated by unpaired Student’s t test. The scatter plots were determined as in Fig.1. The proportion of Ly6C^+^, B220^+^ cells present in Flt3L treated young and aged mice splenocytes is depicted in B and a comparison of young and aged pDC that are CD11c^+^, PDCA-1^+^ and CCR9^+^ was made using overlay histograms (C). The shaded histograms indicate young splenic pDC and the solid line indicates aged pDC. The results depicted in this figure were reproduced in 2 separate experiments.

Since the aged mouse intestine contains fewer pDC than the young mouse intestine, irrespective of LPS treatment (Figure 1d), it is possible that pDC do not emigrate from the bone marrow to the intestine in aged mice or that aged pDC do not migrate as efficiently as young pDC. Therefore, both aged B6 mice and CD45.1 young mice were treated with Flt3L for 10 days, splenocytes harvested and phenotyped for proportion of B220^+^, Ly6C^+^ pDC. The aged splenocyte population was labeled with the vital dye CFSE. An equal number of young CD45.1^+^ pDC and aged CFSE-labeled aged pDC were mixed and then transferred into young CD45.2^+^ LPS-treated hosts. The proportion of injected young CD45.1^+^ and CFSE-labeled aged pDC that was recovered in the epithelial layer 18 hours later was quantified. Figure 6a shows that significantly fewer aged pDC compared to young pDC accumulated in the young host intestine. A possible mechanism for the failure of aged pDC migration can be inferred by further phenotypic analysis of the Ly6C^+^, B220^+^ pDC in the Flt3L-treated splenocyte preparations. As shown in Fig. 6B and C). The aged CFSE-labeled pDC population was more heterogeneous as far as expression levels of CD11c and PDCA-1 compared to the young CD45.1^+^ pDC population and slightly lower expression of CCR9 was noted on aged pDC (Fig. 6C). It is not known whether the expression of CD11c or PDCA-1 might affect migration of pDC or whether a slight decrease in CCR9 expression could account for the dramatic difference in accumulation of aged CFSE-labeled pDC in the young host intestinal epithelial layer. Nevertheless, our data show that the decreased number of pDC in the aged mouse intestine is related to both the altered microenvironment of the aged intestine with suboptimal expression of CCL25 (Fig. 2) and intrinsic defect(s) in aged pDC migratory capacity (Fig. 6).

## DISCUSSION

pDCs are sentinel immune cells. They comprise a unique DC subset that induces rapid host defense through type I IFN-dependent pathways [38], which feed in to both innate and adaptive cascades [6]. Their indispensability to immune competence is highlighted by experimental studies using conditional pDC deletion system that shows pDC-dependent survival from burns [39] and by survival studies in experimental bacterial sepsis [40]. In human health, pDC functional impairments have been linked to poor outcomes of disease such as in the settings of sepsis [41-43], coronary artery disease [44], and many autoimmune diseases [45, 46].

The gastrointestinal mucosa is a unique microenvironment wherein pDCs must distinguish pathogen-associated danger signals from signals associated with resident commensal microflora [47]. Inasmuch as pDCs themselves have direct bactericidal activity [48] and serve as biological bridges between innate and adaptive immunity, we examined how aging affects the migration of intestinal pDCs, alterations thereof could help explain reduced efficiency of mucosal immunity with aging [1]. Here, we adopted the classic LPS endotoxemia model that had been employed previously to show the role of pDC in conventional mucosal immunity in young adult mice [38]. Indeed, our data show age-related decreases in absolute numbers of iIEC and pDC (Fig. 1). After LPS administration, there were decrements in the proportions and absolute numbers of pDC in both young and aged mice, even though the expression of CCL25 (a specific ligand for CCR9 expressed on pDC) was notably increased in young mice but was correspondingly decreased in aged mice. In addition, examination of iLC revealed an increased proportion of pDC in the LPS-treated mice compared to the PBS-treated mice, suggesting likely pDC extravasation into the lumen contributes to the observed reduced levels of pDC in the epithelial layer after an inflammatory LPS stimulus (Fig. 4). Indeed, the adoptive transfer studies showed aged pDC did not accumulate in the host intestinal epithelial layer as efficiently as transferred young pDC. Thus, the mucosal immune system in aged mice could be compromised with an intrinsic inability of aged pDC to migrate to the intestine, as well as to decreased expression of CCL25 that is normally required to attract pDC.

The low total numbers of iIEC in aged mice was expected since there was notable decrease in the number of cells recovered from other mucosal associated lymphoid tissues in aged mice, including the Peyer’s Patch and mesenteric lymph node [1]. Dysregulation of hematopoiesis, including stem cell intrinsic defects and bone marrow niche cell defects [49, 50] and thymic atrophy [51, 52] during aging are likely contributing factors for the failure to populate the immune system depots in aged mice to the same extent as young mice. The failure to mobilize the same percentage of pDC in the spleens of Flt3L-treated aged compared to young mice (Fig. 6B) also attests to hematopoietic deficiencies in the aged mouse.

Since enhanced production of inflammatory mediators in response to LPS endotoxemia has been noted in the aged mouse [31, 32, 53], we compared the mRNA expression of the chemokines *CCL2* and *CCL25* as well as inflammatory mediators *IL-6*, *iNOS*, *IFNγ* and *RAGE* (Fig. 3). After LPS administration, significant increases in *CCL2* mRNA were observed in both young and aged mice compared to PBS-treated mice. We also found increases in *IL-6*, *iNOS* and *RAGE* mRNA levels in aged LPS-treated mice, although only *IL-6* levels were statistically significant from that of young LPS-treated mice. We should note that given the low dose of LPS used (2.0mg/kg) and the time of harvesting samples (18 hours post-LPS), it is possible that elevations of specific transcripts could require higher doses and/or detection at earlier time periods. But irrespective of transcription kinetics, our remarkable finding is that the *CCL25* mRNA level was significantly decreased in the aged LPS-treated mice compared to young LPS- treated mice, an observation that parallels the CCL25 protein levels (Fig. 2). In contrast, the elevated level of CCL25 protein seen in young LPS-treated mice does not parallel the levels of *CCL25* mRNA seen in PBS- versus LPS-treated young mice. Whether this decreased *CCL25* mRNA level is due to transcript instability or to reduced half-life of the protein is unknown. While the relevant *cis*-acting regulatory elements that drive *CCL25* gene transcription have been identified [35], it also remains to be examined whether the efficiency of these transcriptional regulatory elements becomes altered with advancing age.

CCL25 expression in the mucosa of mice has been shown to increase after TNF-α administration with corresponding increased lymphocyte adhesion in the lamina propria [54]. Here, we examined whether the changes in CCL25 expression in young and aged mice would affect migration of adoptively transferred pDC obtained from Flt3L-treated CD45.1 mice. The data show that the proportion of adoptively transferred pDC within the endogenous CD45.1^+^ pDC population of LPS-treated young mice increased compared to young PBS-treated mice (Fig. 5C), a finding that was not observed in similar adoptive transfer experiments with LPS- versus PBS-treated aged mice (Fig. 5C). Both the absolute number of CD45.1^+^ pDC recovered, and the % of injected pDC recovered showed significant decreases in aged LPS-treated mice compared to young LPS-treated mice (Fig. 5E and F). Clearly, fewer aged pDC compared to young pDC migrated to the intestines of young mice (Fig. 6A) and young pDC migrated more efficiently to the young mouse intestine in contrast to the aged mouse intestine (Fig. 5).

Several investigators have shown that mucosal DC can sample gut luminal contents by penetrating epithelial cell tight junctions [55]. CX3CR1^+^ lamina propria DC can form transepithelial dendrites and sample commensal and pathogenic bacteria in the lumen [56]. CX3CR1^+^ cells are local residents that do not migrate to draining mesenteric lymph nodes [57]. Such ability of lamina propria-resident DC to sample gut luminal contents has been linked to TLR signaling of intestinal epithelial cells [58]. In addition to sampling of luminal contents, certain DC subpopulations (along with other cells) move into the gut lumen. In a model of direct administration of non-invasive *Salmonella* into small intestinal loops, *Salmonella*-containing DCs were found in the lumen [59]. The appearance of cells in the lumen after LPS administration was not a result of sloughing of the epithelial layer since histological analysis of small intestinal tissue from both PBS and LPS-treated young and aged mice showed an intact epithelium (unpublished observations). These observations suggest that iLCs such as those we report here (Fig. 4) could be vital to host defense by facilitating detection and elimination of luminal pathogens. Indeed, luminal pDC have been reported in the lungs where such cells have been shown to play a role in the prevention of apoptosis of mucosal T cells [60].

In the present work, we found that the absolute number of iLCs recovered by simply flushing the intestinal lumen was very small and variable, an observation that is probably not surprising considering intestinal peristalsis. Nevertheless, the iLCs recovered contained CD3^+^ and CD3^-^ populations similar to the iIEC population, except for a larger proportion of CD3^-^ luminal cells (Fig. 4). These CD3^-^ iLCs may represent a recruited population of cells as indicated by the increased proportion of pDC in the iLCs from the lumen of LPS-treated young mice compared to young-PBS treated mice (Fig. 4D).

Analysis of iLCs from mice that had received adoptively transferred CD45.1^+^ pDC revealed that an increase in CD45.1^+^ cells was seen in this population after LPS administration (Fig. 5G). This indicates that pDC can migrate from the blood to the epithelial layer and finally into the lumen. In contrast, very few iLCs were recovered from the intestinal lumens of aged mice (Fig. 4), which was expected since they have an overall fewer number of iIEC than young mice (Fig. 1). This suggests that if the luminal population plays a role in host defense, aged mice would also be at a disadvantage, since the lower number of iIEC in aged mice would presumably result in corresponding fewer iLC moving into the lumen.

If the intestinal milieu alone governs the migration of pDC, then young and aged pDC should migrate to the young, inflamed intestine equally. Here we compared the migratory capacity of young versus aged pDC by combining an equal number of young and aged pDC and adoptively transferring them into an LPS-treated young mouse (Figure 6). Significantly fewer aged Ly6C^+^, B220^+^ pDC than young pDC were recovered from the intestinal epithelial layer. However, the Ly6C^+^, B220^+^ aged pDC were more heterogeneous for the expression of CD11c and PDCA-1 and expressed lower CCR9 level than young pDC. The effect of these differences in phenotype on pDC migration is unknown but the decreased number of pDC in the epithelial layer of aged mice (Fig. 1D) is consistent with our data on intrinsic lack of aged pDC migratory capacity (Fig. 6A) as well as a decrease in CCL25 expression (Fig. 2).

In summary, our data show age-related defects or inefficiencies in intestinal pDC function. Along with an overall decrease in their numbers, aged pDCs have an intrinsic migratory defect either to the intestinal epithelium or to the lumen following administration of LPS. Since pDCs are an important link between the innate and adaptive immune system as direct sensors of pathogenic stimuli [61], and as activators of myeloid DC migration from the intestinal lamina propria to the mesenteric lymph nodes [23], age-related defects of intestinal pDC migration may profoundly affect host defense. Of interest will be to examine how aged pDCs respond to parallel changes in the intestinal microflora with aging [62] and contribute to increased incidence of gastrointestinal disorders in old age.

## Supplementary Materials

The Supplemenantry data can be found online at: www.aginganddisease.org/EN/10.14336/AD.2021.0119.


