# Supplementary material for: Disparate Recruitment and Retention of Plasmacytoid Dendritic Cells to The Small Intestinal Mucosa between Young and Aged Mice
Source: Aging Dis. 2021 Aug 1;12(5):1183–96. doi: 10.14336/AD.2021.0119 (PMC8279532; doi:10.14336/AD.2021.0119)
Supplement: Supplementary file 1 [file AD-12-5-1183-s.pdf]

SUPPLEMENTARY DATA

Disparate Recruitment and Retention of Plasmacytoid Dendritic Cells to The Small Intestinal Mucosa between Young and Aged Mice

Rosemary A. Hoffman[1,2,*], Sulan Huang[3], Geetha Chalasani[4], Abbe N. Vallejo[5,6,*]

© 2021. Hoffman RA et al. Published online at http://www.aginganddisease.org/EN/10.14336/AD.2021.0119

SUPPLEMENTARY DATA

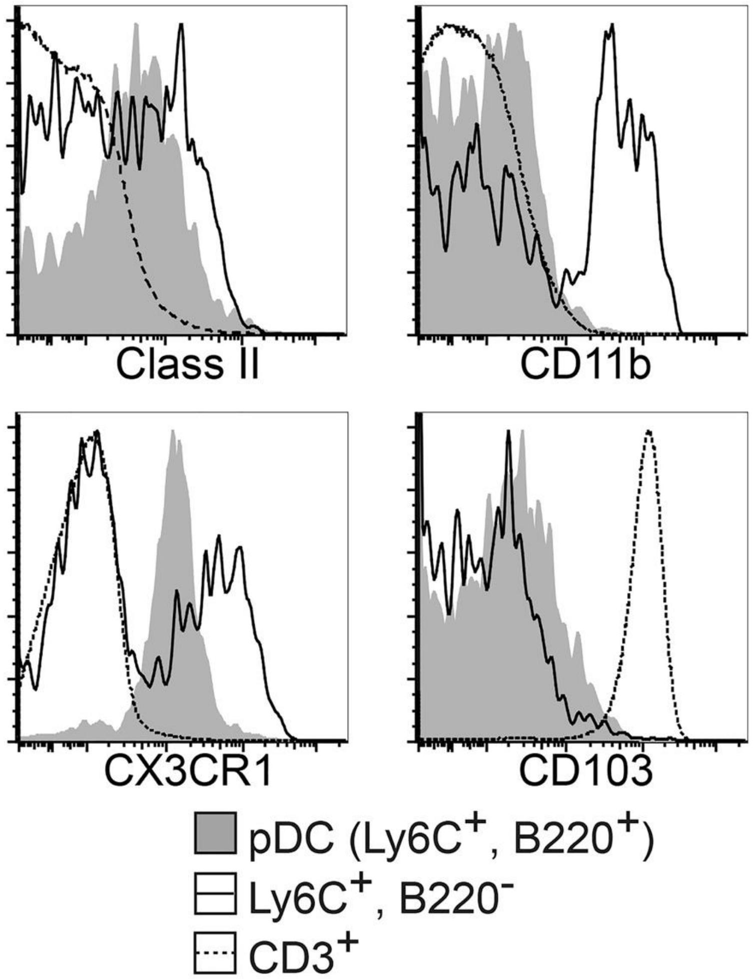

Supplemental Figure 1. Additional phenotypic analysis of pDC within the iIEC population. Ly6C^{+}, B220^{+} pDC in the iIEC of a young B6 mouse were further phenotyped for class II, CD11b, CX3CR1, and CD103. The pDC are represented by the shaded areas in the histograms, Ly6C^{+}, B220^{-} population is indicated by a solid line and CD3^{+} population is indicated by the dotted line. CX3CR1-GFP$^{+/-}$ mice were used to detect CX3CR1 expression. The histograms are representative of 3 experiments.

© 2021. Hoffman RA et al. Published online at http://www.aginganddisease.org/EN/10.14336/AD.2021.0119

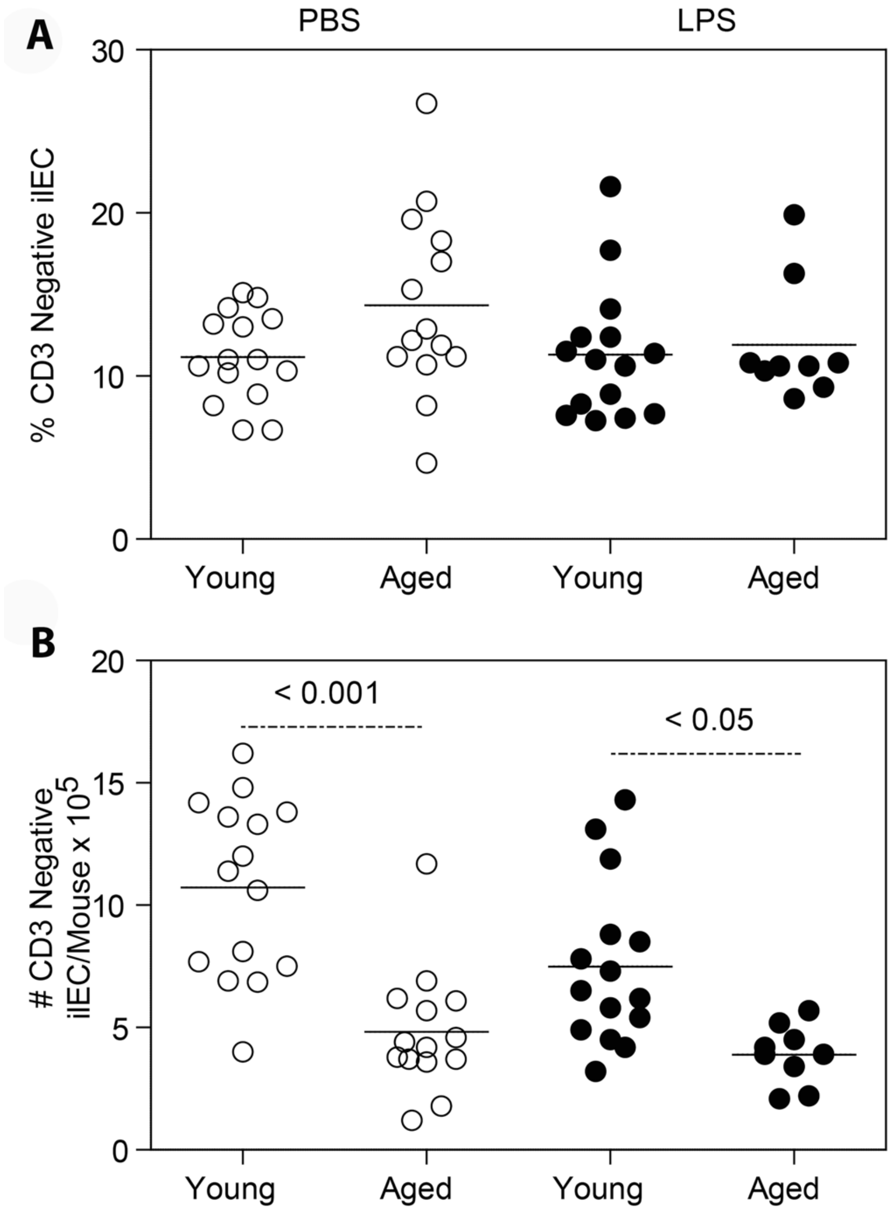

Supplementary Figure 2. Proportion and absolute number of CD3⁻ iIEC. The proportions (**A**) and absolute numbers (**B**) of CD3⁻ iIEC from young and aged mice injected with PBS or LPS are depicted. Scatter plots and P-values were determined as in Figure.1.

© 2021. Hoffman RA et al. Published online at http://www.aginganddisease.org/EN/10.14336/AD.2021.0119

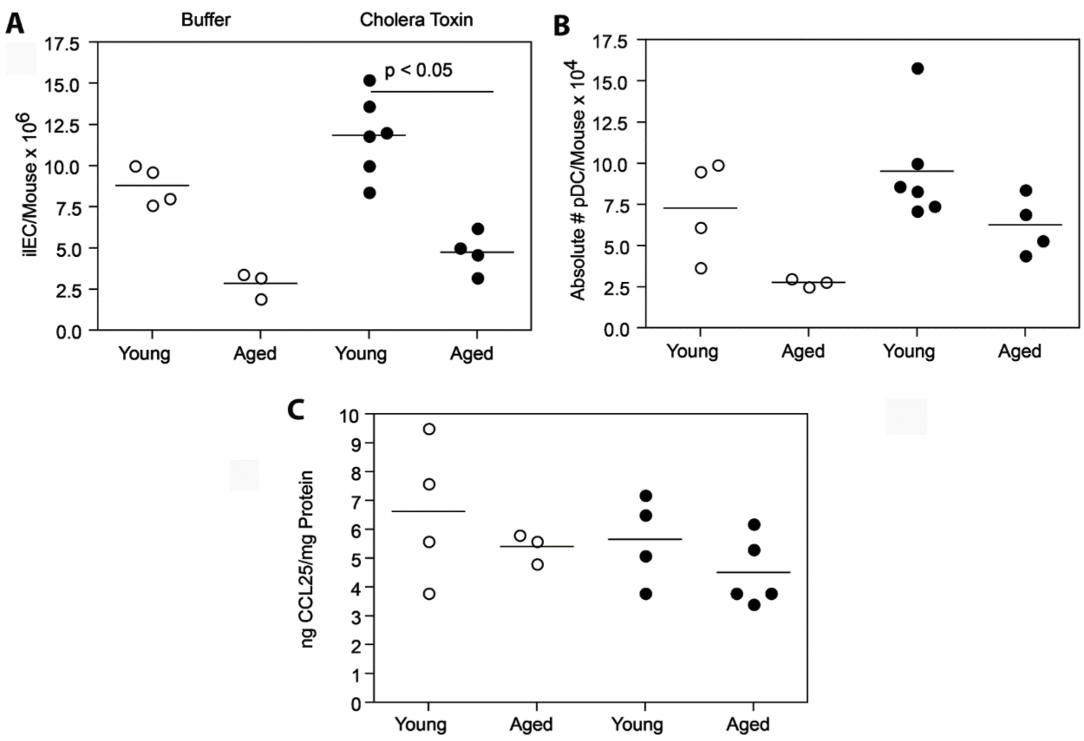

Supplementary Figure 3. Characterization of intestinal mucosa after oral administration of cholera toxin. The absolute number of iIEC (**A**), pDC (**B**) and CCL25 protein levels (**C**) in the intestinal mucosa of young and aged mice 2-3 hours after receiving an oral gavage of 10ug cholera toxin in 0.3 ml of carbonate buffer or buffer alone. Scatter plots and P-values were determined as in Figure 1.

© 2021. Hoffman RA et al. Published online at http://www.aginganddisease.org/EN/10.14336/AD.2021.0119

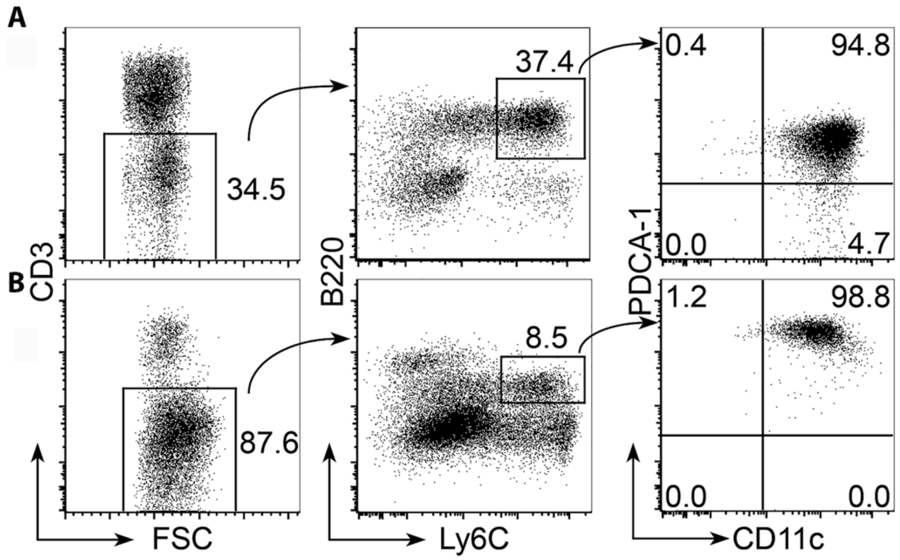

Supplementary Figure 4. Phenotype of iIEC and iLC recovered from Flt3L-treated mouse. iIEC (**A**) and iLC (**B**) were recovered from a Flt3L-treated young mouse and the CD3 negative population was phenotyped for Ly6C, B220, CD11c and PDCA-1. This analysis of intestinal cells from Flt3L-treated mice is representative of 2 separate experiments.

© 2021. Hoffman RA et al. Published online at http://www.aginganddisease.org/EN/10.14336/AD.2021.0119
